# Botulinum neurotoxin: from molecular pathogenesis to emerging countermeasures

**DOI:** 10.1007/s44446-026-00090-2

**Published:** 2026-06-02

**Authors:** Sultan Almudimeegh

**Affiliations:** Department of Pharmacology and Toxicology, College of Pharmacy, King Saud University, Riyadh, Saudi Arabia

**Keywords:** Botulism, SNARE, SNAP-25, BoNT, Clostridium botulinum, Neuroparalysis, PROTAC

## Abstract

Botulism is a severe neuroparalytic syndrome caused by botulinum neurotoxins (BoNTs)—among the most potent biological agents—with an estimated human lethal dose (LD_50_) of approximately 1 ng/kg. By cleaving soluble N-ethylmaleimide–sensitive factor attachment protein receptor (SNARE) proteins at cholinergic nerve terminals, BoNTs block acetylcholine release, producing potentially fatal flaccid paralysis. Despite advances in supportive care, current therapeutic options remain limited to antitoxin administration, which is effective only against circulating toxin and cannot neutralize intracellular BoNT. Consequently, patients often require prolonged mechanical ventilation and rehabilitation. To address these limitations, this review provides a comprehensive overview of botulism, spanning historical recognition, structural and mechanistic insights into BoNT activity, clinical manifestations, and current treatment strategies, while highlighting therapeutic gaps. Particular emphasis is placed on emerging preclinical interventions, including small-molecule inhibitors, antibody-based therapeutics, intracellular clearance strategies, and gene- and RNA-based modalities, reflecting rapid progress in structural biology and pharmacology. Collectively, these advances highlight both the promise and the remaining translational challenges of developing next-generation countermeasures, with implications for clinical management and biodefense preparedness.

**Graphical Abstract:**

*Botulism: Pathophysiology, Clinical Presentation, and Therapeutic Landscape.* Schematic overview of botulism from exposure to treatment. BoNT exposure may occur through foodborne, infant, wound, iatrogenic, or bioterror-related routes. After systemic distribution, the toxin binds peripheral cholinergic nerve terminals, undergoes endocytosis, and translocates its light chain into the cytosol, where SNARE cleavage blocks acetylcholine release. This results in the characteristic descending paralysis of botulism, with early cranial nerve involvement followed by limb weakness and respiratory failure. The figure also highlights the limited window for antitoxin efficacy before neuronal internalization and summarizes both current therapies and emerging preclinical countermeasures aimed at neutralizing circulating toxin, enhancing intracellular clearance, or restoring neuronal function. *ACh, acetylcholine; BIG-IV, botulism immune globulin intravenous; BoNT, botulinum neurotoxin; HBAT, heptavalent botulism antitoxin; IV, intravenous; PROTAC, proteolysis-targeting chimera; RNA, ribonucleic acid; SNAP-25, synaptosomal-associated protein 25; SNARE, soluble N-ethylmaleimide–sensitive factor attachment protein receptor; VAMP, vesicle-associated membrane protein (synaptobrevin)*

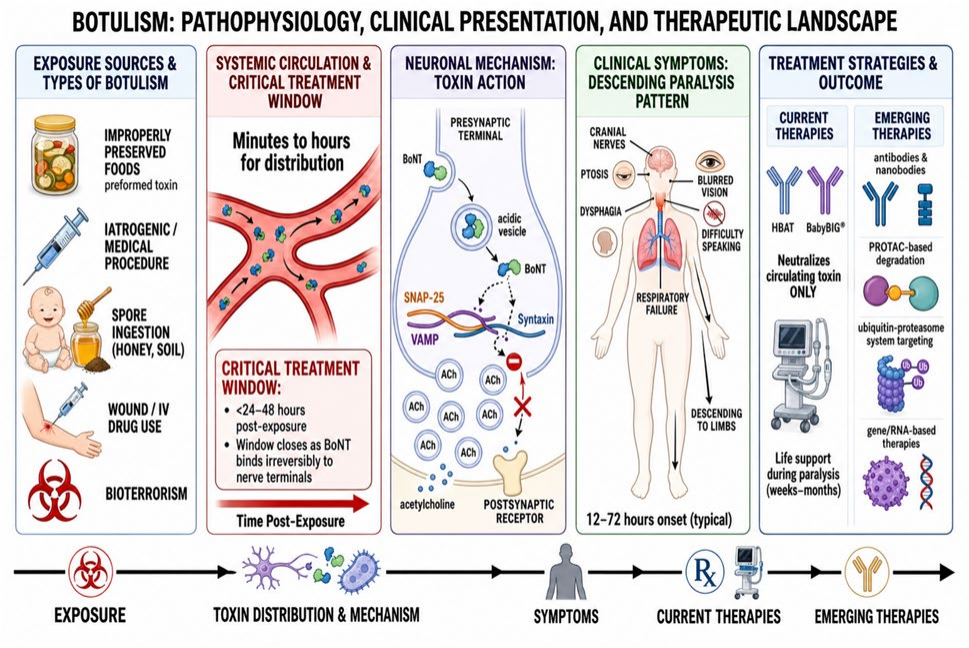

## Introduction

Botulinum neurotoxins (BoNTs) occupy a unique position at the intersection of toxicology and therapeutics. On one hand, these bacterial proteins have become valuable tools in clinical neurology and cosmetic medicine. On the other hand, they remain best known as the causative agents of botulism. This duality reflects the extreme precision of their molecular mechanism: by selectively targeting peripheral cholinergic synapses, BoNTs can produce either therapeutic benefits or devastating systemic paralysis, depending on the context of exposure. These neurotoxins are produced primarily by the anaerobic, Gram-positive bacterium *Clostridium botulinum (C. botulinum)*. Clinical manifestations arise from inhibition of acetylcholine (ACh) release at the neuromuscular junctions (NMJ) and autonomic synapses (Habermann And Dreyer 1986; Jahn And Niemann 1994). Clinically, botulism presents as a descending flaccid paralysis accompanied by ptosis, dysphagia, dysarthria, and autonomic dysfunction (e.g., dry mouth, constipation, and tachycardia), potentially progressing to respiratory failure (Rao et al. 2021). The only approved specific therapy is botulinum antitoxin, which should be administered as early as possible, ideally within the first 24–48 h after symptom onset. Patients often require prolonged intensive care and mechanical ventilation because of the prolonged intracellular persistence of the toxin light chain (Al-Saleem et al. 2011; Patel et al. 2022; Tacket et al. 1984).

The condition now known as botulism was first described clinically at the beginning of the nineteenth century in Germany, where Justinus Kerner characterized a set of symptoms in 76 people as a descending flaccid paralysis after eating spoiled smoked sausage. He called it the “sausage poison” (Erbguth 2004). In 1870, John Müller introduced the word "botulism", derived from the Latin term for sausage, *botulus* (Erbguth And Naumann 1999). A quarter of a century later, a similar outbreak took place in Ellezelles, Belgium, where individuals exhibited the same symptoms. Emile van Ermengem isolated the bacterium responsible for botulism from the smoked ham and named it *Bacillus botulinus*. The bacterium was later renamed *C. botulinum* (Cherington 1998).

Botulism represents a significant threat to public health. Untreated or delayed treatment of botulism cases often requires long-term ICU admission and mechanical ventilation for several months (Arnon et al. 2001). Widespread outbreaks and epidemics can overwhelm healthcare systems (Newkirk And Hedberg 2012). At the individual level, patients may experience intense physical challenges, including extreme muscle weakness (quadriparesis), visual impairment due to ptosis (drooping eyelids), impaired speech and difficulty breathing. Psychologically, the abrupt decline can trigger shock, agitation, or depression, often accompanied by uncertainty. Botulism frequently leads to long-lasting effects that impact quality of life even after hospital discharge, necessitating extensive rehabilitation (Lonati et al. 2020; Cohen And Anderson 1986). Therefore, early treatment is critical to prevent these serious consequences. The objective of this review is to provide an integrated overview of botulism, including its history, molecular mechanisms, clinical presentation, diagnosis, and current treatment strategies. It also examines emerging preclinical countermeasures designed to neutralize BoNT after neuronal internalization and highlights the main translational challenges and future opportunities for next-generation therapies in clinical care and biodefense.

## Pathophysiology and disease course

### Clostridium botulinum

The Gram-positive, spore-forming anaerobic bacterium *C. botulinum* produces BoNT, which is recognized as one of the most potent biological substances known. The bacterium maintains its environmental persistence through its ability to form metabolically dormant endospores, which allow it to survive extreme temperatures, desiccation, and chemical exposure. The spores of *C. botulinum* are ubiquitous in soil and aquatic sediments, as well as in improperly processed food products, posing an ongoing threat to public health and food safety (Arnon et al. 2001; Li et al. 2025).

All *C. botulinum* strains belong to four phylogenetic groups (I–IV), which exhibit different genetic and physiological characteristics, yet they all produce BoNT as a common trait. Human botulism-causing strains belong to Groups I and II, while Groups III and IV primarily affect animals (Rawson et al. 2023). The bacterium requires strict anaerobic conditions, along with specific physicochemical parameters, such as a low redox potential and an optimal pH of 4.6 or higher, as well as sufficient nutrient availability, to germinate and produce the toxin (Bilska et al. 2024). The transcription of the neurotoxin gene cluster is controlled by the *botR* gene, a key regulatory element. The organism’s ability to thrive as a saprophyte, combined with its remarkable resilience, has contributed to its evolutionary success and medical importance (Rawson et al. 2023).

Infection with *C. botulinum* or exposure to its toxin results in several clinical forms of botulism, which are classified based on the route of exposure and the site of toxin production. Historically, foodborne botulism, one of the most prevalent forms, develops after individuals consume food contaminated with preformed BoNTs. Improper processing or preservation of foods under anaerobic conditions leads to this form of botulism (Le Marechal et al. 2025). In wound botulism, wounds become contaminated with *C. botulinum* spores through exposure to soil or, increasingly, the use of contaminated injection drug equipment; the spores then germinate under anaerobic conditions and produce toxin in vivo (Edwards et al. 2022; Elrayes et al. 2024). Infant botulism occurs when ingested spores germinate into BoNT-producing vegetative cells in the underdeveloped infant gut microbiome (Weiss et al. 2025). The popularity of Botox for cosmetic procedures and its growing medical applications have contributed to the rise of iatrogenic botulism. This can result from the accidental overdose or incorrect injection of therapeutic BoNT (An et al. 2025). Another extremely rare form is exposure to aerosolized BoNT, typically associated with laboratory incidents or bioterrorism (Snow et al. 2021). Regardless of exposure route, all types of botulism share the same fundamental pathomechanism: BoNTs selectively target cholinergic neurons of the somatic and parasympathetic nervous systems, and the intracellular persistence of the toxin light chain contributes to prolonged neuroparalytic symptoms (Cherington 1998).

### Structure and types of botulinum neurotoxins

**Fig. 1:**
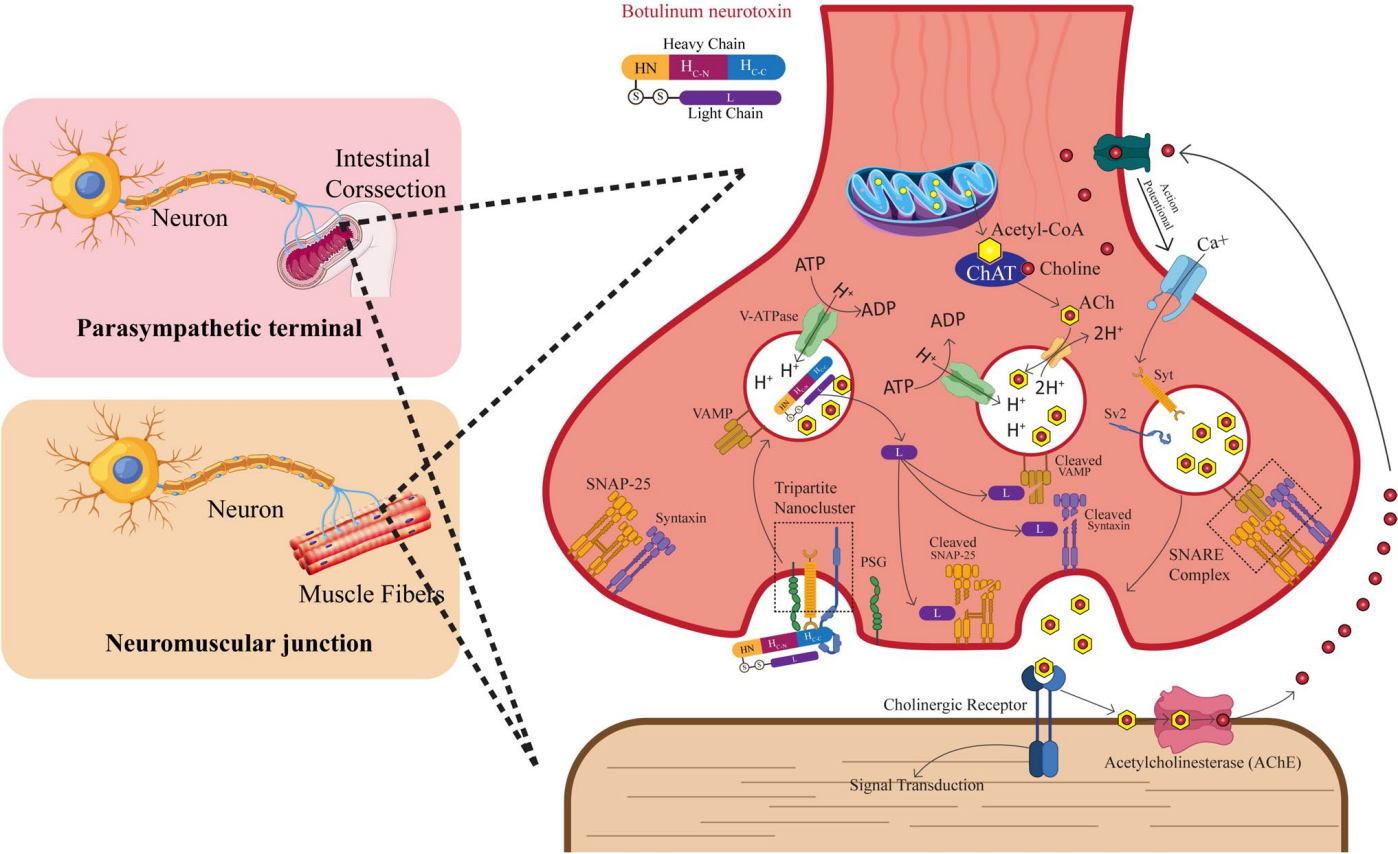
Pathophysiological manifestations of botulism. By preventing acetylcholine release at neuromuscular and parasympathetic cholinergic synapses, BoNT produces a characteristic descending, symmetrical flaccid paralysis accompanied by autonomic dysfunction. The figure illustrates the staged clinical progression of botulism, beginning with early bulbar and cranial nerve involvement, followed by descending paralysis with respiratory muscle compromise, and later autonomic manifestations affecting the gastrointestinal and cardiovascular systems. Corresponding management actions are shown alongside each stage, emphasizing early antitoxin administration, mechanical ventilation for respiratory failure, and supportive care for autonomic instability. *ACh, acetylcholine; BoNT, botulinum neurotoxin; GI, gastrointestinal*

**Fig. 2:**
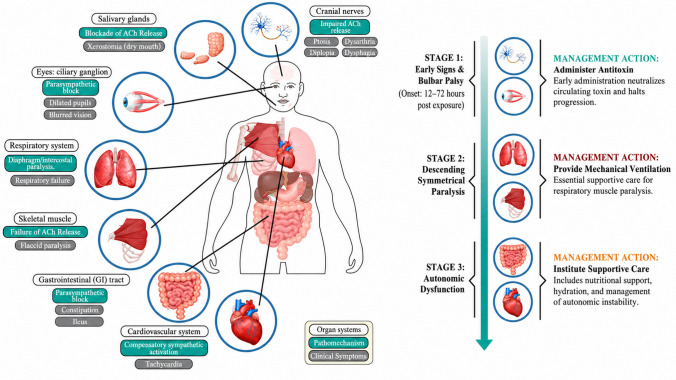
Molecular mechanism of botulinum neurotoxin intoxication at cholinergic synapses. BoNT binds presynaptic receptors, undergoes endocytosis, and translocates the light chain, which cleaves SNARE proteins and prevents acetylcholine release. This occurs at both neuromuscular junctions (causing flaccid paralysis) and parasympathetic cholinergic synapses (causing autonomic dysfunction such as dry mouth, constipation, and pupillary abnormalities). *ACh, acetylcholine; AChE, acetylcholinesterase; ADP, adenosine diphosphate; ATP, adenosine triphosphate; Ca²⁺, calcium ion; ChAT, choline acetyltransferase; CoA, coenzyme A; H⁺, hydrogen ion (proton); HN, heavy-chain N-terminal translocation domain; HC-N, N-terminal subdomain of the heavy-chain C-terminal receptor-binding domain; HC-C, C-terminal subdomain of the heavy-chain C-terminal receptor-binding domain; L, light chain; PSG, polysialoganglioside; S–S, disulfide bond; SNAP-25, synaptosomal-associated protein 25; SNARE, soluble N-ethylmaleimide–sensitive factor attachment protein receptor; SV2, synaptic vesicle glycoprotein 2; Syt, synaptotagmin; V-ATPase, vacuolar-type proton ATPase; VAMP, vesicle-associated membrane protein (synaptobrevin)*

**Fig. 3:**
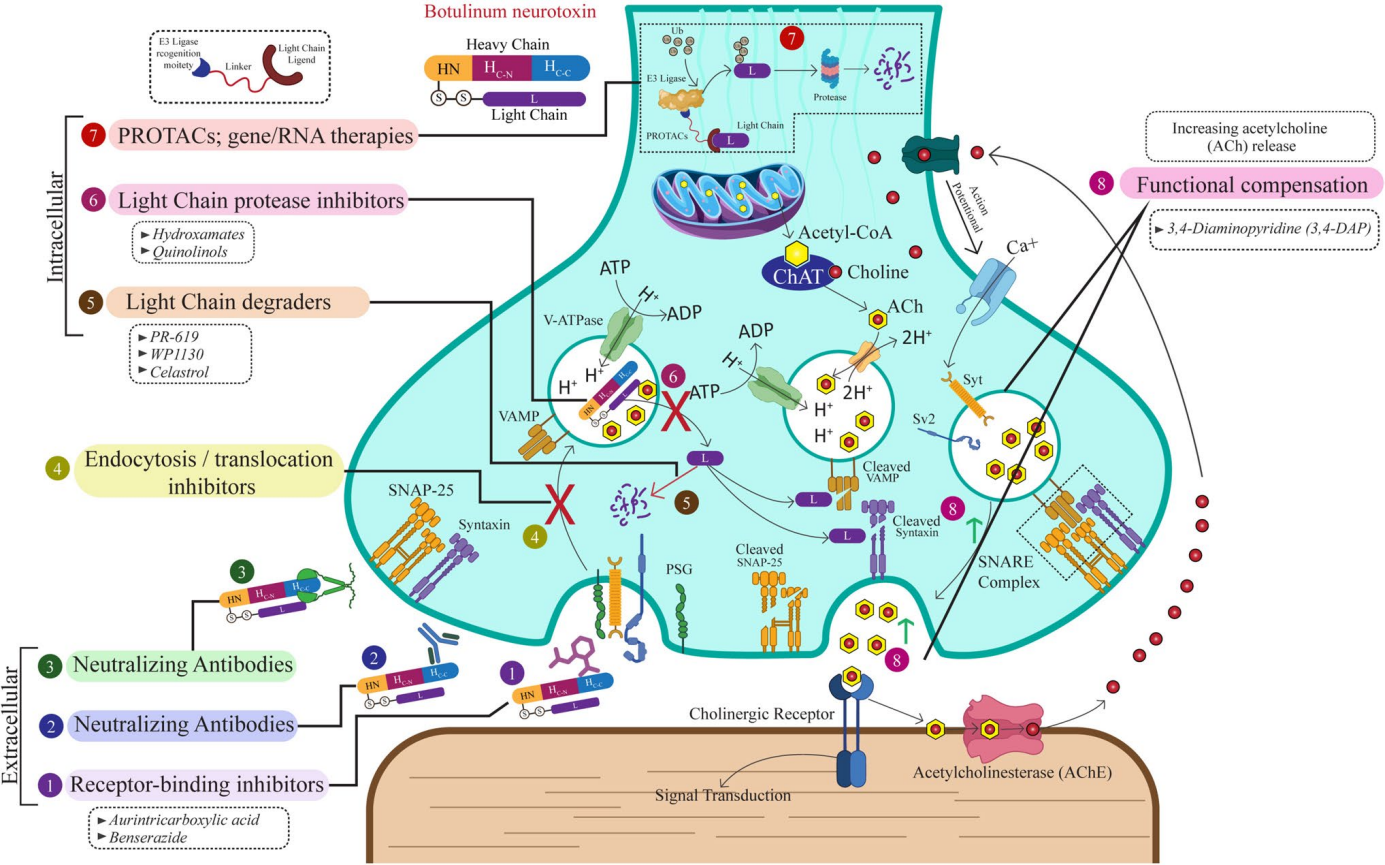
Sites of therapeutic intervention against botulinum neurotoxin. Strategies target extracellular binding, endosomal trafficking, intracellular protease activity, or clearance of the light chain, while functional approaches such as 3,4-DAP enhance acetylcholine release. Eight intervention points are illustrated, spanning receptor-binding inhibitors (1), neutralizing antibodies targeting free and receptor-bound toxin (2–3), endocytosis/translocation inhibitors (4), light chain degraders (5), light chain protease inhibitors (6), PROTACs and gene/RNA therapies (7), and functional compensation via 3,4-DAP (8). These therapies aim to restore transmission at both neuromuscular and parasympathetic synapses. *3,4-DAP, 3,4-diaminopyridine; ACh, acetylcholine; AChE, acetylcholinesterase; ADP, adenosine diphosphate; ATP, adenosine triphosphate; Ca²⁺, calcium ion; ChAT, choline acetyltransferase; CoA, coenzyme A; H⁺, hydrogen ion (proton); HC-C, heavy chain C-terminal receptor-binding subdomain; HC-N, heavy chain C-terminal translocation subdomain; HN, heavy chain N-terminal domain; L, light chain; PSG, polysialoganglioside; PROTAC, proteolysis-targeting chimera; RNA, ribonucleic acid; S–S, disulfide bond; SNAP-25, synaptosomal-associated protein 25; SNARE, soluble N-ethylmaleimide–sensitive factor attachment protein receptor; SV2, synaptic vesicle glycoprotein 2; Syt, synaptotagmin; Ub, ubiquitin; V-ATPase, vacuolar-type proton ATPase; VAMP, vesicle-associated membrane protein (synaptobrevin)*

BoNTs are single-chain polypeptides of approximately 150 kDa. They can be enzymatically cleaved into two functionally separate domains: the light chain (LC) and the heavy chain (HC). These are connected by a disulfide bond that is crucial for the toxin’s activity. The LC (50 kDa) is a zinc-dependent metalloprotease that targets soluble N-ethylmaleimide-sensitive factor attachment protein receptor (SNARE) proteins, which are required for synaptic vesicle exocytosis, thereby interfering with ACh release at cholinergic nerve terminals, including NMJ and autonomic synapses. The HC (100 kDa) consists of two subdomains: the N-terminal domain, responsible for translocating the LC into the cytosol after endocytosis, and the C-terminal domain, which mediates high-affinity binding to neuronal receptors, including gangliosides and synaptic vesicle proteins such as SV2 or synaptotagmin (Kosenina et al. 2021). Figure 1 summarizes the sequential steps of intoxication and the toxin’s action at somatic and autonomic cholinergic synapses. As illustrated, intoxication begins with HC binding to the presynaptic membrane, followed by endocytosis, LC translocation into the cytosol, SNARE cleavage, and blockade of ACh release. Building on this molecular framework, Fig. 2 (discussed in the Disease Course section) depicts the downstream clinical consequences—the descending pattern of paralysis and autonomic dysfunction—that arise when SNARE cleavage disrupts neurotransmission across cranial and peripheral motor neurons. Together, Figs. 1 and 2 thus link the molecular mechanism to the clinical phenotype, providing the conceptual basis for the therapeutic landscape illustrated in Fig. 3.

There are seven serotypes of BoNT (A through G), identified by their unique antigenic characteristics and the specific SNARE proteins they cleave (Johnson And Montecucco 2008; Schiavo et al. 2000). Types A, B, E, and F are linked to human botulism, while types C and D mainly affect animals, and type G is rarely encountered. BoNT/A and BoNT/E both cleave soluble N-ethylmaleimide-sensitive factor attachment protein receptor 25 (SNAP-25) at different sites, whereas BoNT/B, D, F, and G cleave vesicle-associated membrane protein (VAMP). BoNT/C uniquely cleaves both SNAP-25 and syntaxin (Tsai et al. 2024; Critchley 1991; Pellett et al. 2019). These variations determine not only receptor specificity but also intracellular persistence and clinical duration. BoNT/A, for instance, is recognized for causing paralysis that endures for several months, unlike BoNT/E, whose effects typically dissipate within weeks. This difference mainly results from the longer intracellular half-life of BoNT/A’s LC within the neuron, due to reduced vulnerability to degradation pathways. BoNT/E’s LC, on the other hand, is more susceptible to the ubiquitin–proteasome system (UPS) and thus has a much shorter half-life (Tsai et al. 2017; Eleopra et al. 1998). These serotype-specific differences in SNARE targets, cleavage sites, intracellular persistence, and clinical relevance are summarized in Table 1.

**Table 1:** Comparative molecular mechanisms of botulinum neurotoxin (BoNT) serotypes: SNARE targets, cleavage sites, and key clinical/functional notes

Serotype	Target SNARE	Cleavage Site/Specificity	Key Clinical/Functional Notes	References
A	SNAP-25	Gln197–Arg198	Most common in human botulism; longest duration of action (several months); widely used therapeutically (e.g., Botox®); LC highly stable intracellularly due to resistance to proteasomal degradation	Tsai et al. 2024; Critchley 1991; Tsai et al. 2017; Eleopra et al. 1998)
B	VAMP/Synaptobrevin	Gln76–Phe77	Common in human botulism (foodborne & wound); shorter duration than A; used clinically (e.g., Myobloc®/NeuroBloc®); more rapid LC degradation	Tsai et al. 2024; Critchley 1991; Pellett et al. 2019; Eleopra et al. 1998)
C	SNAP-25 and Syntaxin	SNAP-25: Arg198–Ala199; Syntaxin: multiple sites	Dual SNARE targeting → broader blockade; rare in humans; mainly veterinary relevance (birds, cattle); longer persistence in some hosts	Tsai, et al. 2024; Critchley 1991; Pellett et al. 2019)
D	VAMP/Synaptobrevin	Distinct site from B/F (specific residue varies by strain)	Primarily affects animals (cattle, birds); very limited human relevance; not a major cause of human botulism	Tsai et al. 2024; Critchley 1991; Pellett et al. 2019)
E	SNAP-25	Arg180–Ile181	Common in foodborne botulism (especially fish/marine products); shortest duration (days to weeks); LC rapidly ubiquitinated and degraded by proteasome	Tsai et al. 2024; Critchley 1991; Tsai et al. 2017; Eleopra et al. 1998)
F	VAMP/Synaptobrevin	Gln58–Lys59	Rare human cases (foodborne); very short persistence; less studied clinically	Tsai et al. 2024; Critchley 1991; Pellett et al. 2019)
G	VAMP/Synaptobrevin	Unique site (distinct from B/D/F)	Extremely rare; very limited clinical data; almost no reported human cases	Tsai et al. 2024; Critchley 1991; Pellett et al. 2019)
X	VAMP1/2/3 (and non-canonical: VAMP4, VAMP5, Ykt6)	Arg66–Ala67 (in VAMP2; homologous sites in others)	Novel serotype; cleaves VAMP at a unique site distinct from B/D/F/G; broadest substrate range among known BoNTs; no reported human cases; low potency in some animal models; potential therapeutic interest; clinical significance under study	Martinez-Carranza et al. 2023; Zhang et al. 2017)

BoNT also occurs in unusual mosaic structures, adding to the complexity of the BoNT family. These structures arise from natural recombination events between different strains of *C. botulinum* that lead to hybrid forms. They incorporate the LC and HC domains typically found in two distinct BoNT serotypes. Consequently, they exhibit a combination of antigenic, functional, and receptor-binding characteristics not found in any single classical serotype. Mosaic toxins frequently display distinct pharmacological and immunological characteristics, complicating diagnostic serotyping and antitoxin effectiveness (Zhang et al. 2011; Moriishi et al. 1996). Recently, a new serotype, designated as BoNT/X, has been identified. This serotype is antigenically unique, cleaving VAMP1–3 at a site targeted by no other BoNT, and does not cross-react with typical antisera; however, its clinical significance remains under study (Martinez-Carranza et al. 2023; Zhang et al. 2017).

### Molecular pathways and mechanisms

The clinical symptoms of botulism result from the action of BoNTs within cholinergic neurons of the peripheral nervous system (PNS). This multi-step process begins with the HC binding to neuronal receptors—gangliosides (e.g., GT1b, GD1a) and proteins such as SV2 or synaptotagmin—depending on the serotype (Black and Dolly 1986; Dolly et al. 1984; Dolly 2003). The physical characteristics of BoNTs and their selective binding ensure the neurotropism of the toxin, confining its action to peripheral cholinergic neurons and thereby explaining both the specificity of the symptoms and the absence of central nervous system involvement, as the toxin does not cross the blood–brain barrier (BBB) (Dolly 2003).

When BoNTs bind to the receptor through the HC, the toxin–receptor complex undergoes clathrin-mediated endocytosis and is taken into acidic endosomes. A conformational shift in the HC’s translocation domain occurs in a low pH environment. This shift induces the formation of a channel in the endosomal membrane that transports the LC from the endosome into the neuron’s cytosol (Dolly et al. 1984; Pantano And Montecucco 2014). The LC then cleaves members of the SNARE complex, which are essential for the fusion of synaptic vesicles with the presynaptic membrane.

The SNARE targets are serotype-specific: BoNT/A and BoNT/E cleave SNAP-25 at distinct sites; BoNT/B, D, F, and G target synaptobrevin/VAMP. BoNT/C cleaves both SNAP-25 and syntaxin. This cleavage halts synaptic vesicle exocytosis and prevents the release of ACh at the NMJ and autonomic synapses. This leads to clinically observable effects: flaccid paralysis and dysfunction of the autonomic nervous system (Tsai et al. 2024; Montecucco and Schiavo 1995).

A key distinguishing feature among BoNT serotypes is the duration of their clinical effects. For example, BoNT/A produces paralysis that can persist for several months, whereas the effects of BoNT/E diminish within a few weeks (Tsai et al. 2024). This difference stems not from varying binding affinities but is fundamentally related to their stability within cells. BoNT/E LC is rapidly ubiquitinated by the E3 ligase TRAF2 and subsequently degraded by the proteasome. In contrast, the LC of BoNT/A is ubiquitinated by HECTD2 but avoids degradation due to the protective role of deubiquitinating enzymes like USP9X and VCIP135. This interplay between ubiquitination and deubiquitination regulates LC stability and the longevity of the toxin (Schiavo et al. 2000; Tsai et al. 2017; Tsai et al. 2010; Eleopra et al. 1998; Foran et al. 2003).

The characteristic descending flaccid paralysis of botulism can be attributed to both structural and physiological factors (Montal 2010). Larger neurons have long, wide axonal terminals with abundant SNARE proteins that may serve as reservoirs for BoNT action, prolonging symptom onset. The toxin’s failure to penetrate the blood–brain barrier, despite its powerful enzymatic activity, is likely attributable to its size (150 kDa) and absence of specialized transport routes into the central nervous system (Kumar And Singh 2025).

Thus, the stepwise journey of BoNT—from receptor binding, vesicular internalization, cytosolic translocation, to SNARE cleavage—underpins the severe and prolonged neuromuscular paralysis seen in botulism. A more comprehensive understanding of these molecular pathways dictates not only the therapeutic applications of BoNT, but also the countermeasures developed against it.

### Disease course and clinical manifestations

The clinical course of botulism illustrates how a microbial toxin can cause rapidly progressive neuromuscular and autonomic dysfunction. It unfolds with a characteristic descending, symmetrical paralysis, accompanied by marked autonomic dysfunction (Huang et al. 2025; Pirazzini et al. 2017). Patients often appear well initially, only to deteriorate alarmingly within hours. BoNT serotypes play a consequential role in determining the severity and duration of clinical symptoms. Typically, recovery from BoNT/A toxicity lasts several months, while recovery from BoNT/E toxicity lasts 2–3 weeks (Rao et al. 2021; Huang et al. 2025). The difference originates from the intracellular stability of the LC: BoNT/A’s LC is more resistant to proteasomal degradation than BoNT/E’s LC, resulting in prolonged SNARE protein cleavage and a slower recovery of synaptic function (Tsai et al. 2017; Tsai et al. 2010; Keller et al. 1999). The hallmark descending pattern of paralysis, accompanied by cranial nerve involvement and autonomic dysfunction, is depicted in Fig. 2. This figure complements Fig. 1 by translating the molecular mechanism of SNARE cleavage into the observable clinical trajectory: beginning with cranial nerve deficits (ptosis, dysphagia) and progressing caudally to respiratory failure, reflecting the anatomical gradient of BoNT vulnerability across different neuronal populations.

The incubation period for botulism typically ranges from 12 to 72 h, though symptoms may not appear for up to 10 days in rare cases, depending on the BoNT dose and serotype (Thirunavukkarasu et al. 2018; Rowlands et al. 2010). The initial period of latency makes it difficult to diagnose the condition because patients first experience non-specific symptoms, including fatigue, vertigo, blurred vision, and dry mouth. However, these nonspecific early symptoms may precede progression to complete respiratory failure (Kumar And Singh 2025).

The main symptom of botulism is a symmetrical, descending flaccid paralysis that differentiates it from other neuromuscular disorders (Rao et al. 2017). The sequential blockade of ACh release at peripheral cholinergic synapses follows a descending pattern, beginning with cranial nerves before advancing to limbs and respiratory musculature. The first signs of the disease appear as ptosis, diplopia, and dysphagia because cranial nerve function is impaired (Rao et al. 2017). These cranial nerve deficits reflect early involvement of bulbar cholinergic neurons—an area of high vulnerability due to its dense cholinergic innervation (Arnon et al. 2001). As the disease evolves, the motor weakness descends symmetrically, involving the upper limbs before the lower limbs. Patients often experience intense difficulties moving their limbs. As paralysis progresses to involve the diaphragm and intercostal muscles, respiratory insufficiency may develop and progress to respiratory arrest. Without mechanical ventilation, death is almost inevitable (Hughes et al. 1981). Respiratory failure remains the leading cause of mortality in botulism (Rao et al. 2021; Cherington 1998; Huang et al. 2025). In severe cases, patients require prolonged ventilatory support for weeks or even months, as the recovery of synaptic function depends on the synthesis and transport of new SNARE proteins and neuronal remodeling (Tsai et al. 2024).

Autonomic symptoms are frequently underappreciated but play a significant role in the clinical picture, especially among mechanically ventilated patients. Severe xerostomia (dry mouth) is one of the earliest and most persistent complaints. It arises from BoNT blockade of ACh release at parasympathetic nerve terminals innervating salivary glands (Manfredi et al. 2011). Severe, treatment-resistant constipation occurs because parasympathetic cholinergic blockade impairs colonic motility (Avril et al. 2024; Couesnon et al. 2012). Tachycardia occurs as a compensatory response to reduced vagal tone, allowing the sympathetic nervous system to dominate. Other symptoms may include blurred vision due to accommodation deficits, pupillary dilation, and orthostatic hypotension (Rao et al. 2021).

It remains unclear why botulism produces symmetrical, descending flaccid paralysis. Cranial motor neurons may be the first to exhibit signs of BoNT exposure because they have an unusually high density of surface receptors (gangliosides and SV2 isoforms) known to bind BoNT and facilitate cellular entry (Rao et al. 2021; Hirokawa And Kitamura 1979). Moreover, these neurons have a relatively limited synaptic reserve and smaller axonal fields, which could contribute to earlier symptom onset. Another contributing factor may be the local concentration of SNARE proteins such as SNAP-25. Cranial neurons may possess lower baseline pools of SNAP-25, meaning that even limited proteolytic cleavage by BoNT can lead to significant neurotransmission failure. Conversely, motor neurons that innervate the limbs and diaphragm, which are impacted at a later stage, may possess more substantial reserves of SNARE proteins or experience slower rates of BoNT absorption, which could postpone the onset of paralysis (Kumar And Singh 2025). Collectively, these anatomical, molecular, and pharmacokinetic elements provide reasonable explanations for the typical descending sequence of paralysis seen in cases of botulism.

One of the most alarming features of botulism is the lucid state in which patients often remain despite their escalating paralysis. Patients remain fully aware of their inability to open their eyes, speak, or move—a profoundly distressing experience. Later in the disease course, a patient may be completely paralyzed yet fully conscious. If mechanical ventilation is not initiated in a timely manner, death is certain (Rao et al. 2021).

### Diagnosis and differential considerations

The diagnosis of botulism is primarily clinical, especially early in the disease course, when prompt treatment is critical (Rao et al. 2021). Laboratory confirmation, typically involving the detection of BoNT in serum, stool, or food samples via a mouse bioassay or PCR-based toxin gene identification, often takes several days and should not delay intervention (Rao et al. 2021; Chimienti et al. 2025). As a result, clinical suspicion based on characteristic findings—especially descending paralysis, autonomic dysfunction, and preserved mental status—is the cornerstone of early diagnosis (Rao et al. 2021). However, these features can be difficult to recognize, particularly when the initial symptoms are vague or attributed to other neurological disorders (Lonati et al. 2020).

Botulism is often misdiagnosed early on, most commonly as myasthenia gravis (MG), especially during a myasthenic crisis. Both conditions can present with ocular symptoms such as ptosis and diplopia, bulbar dysfunction, generalized muscle weakness, and respiratory compromise (Kobaidze And Wiley 2023). At a mechanistic level, the similarity arises from a shared final pathway—ACh transmission failure at the NMJ. In MG, this results from autoantibody-mediated impairment or loss of nicotinic ACh receptors, whereas in botulism, ACh release is inhibited by BoNT-mediated cleavage of SNARE proteins. Despite different mechanisms upstream, the result is the same: failure of cholinergic neurotransmission at the NMJ (Kobaidze And Wiley 2023).

However, subtle distinctions exist. Botulism typically manifests as a symmetrical, descending flaccid paralysis. MG may present more variably and may not be strictly descending. Severe dry mouth, unreactive pupils, and constipation can be features of botulism; these autonomic features are not seen in MG (Sobel 2005). Tests such as repetitive nerve stimulation help to make the distinction but have overlapping findings. Most importantly, the edrophonium test (Tensilon) used in diagnosing MG is not useful in botulism and can be misleading (Sobel 2005). As antitoxin treatment must not be delayed, therapy is often initiated on clinical suspicion alone, particularly during outbreaks or in progressive cranial nerve dysfunction with autonomic signs.

While the diagnosis of botulism is largely clinical, confirmatory testing is essential for epidemiological purposes and definitive case classification (Rao et al. 2021; Sobel 2005). The gold standard remains the mouse bioassay, though more rapid and humane alternatives such as enzyme-linked immunosorbent assay (ELISA) and polymerase chain reaction (PCR)-based assays targeting BoNT genes are increasingly used. These tests can detect BoNT in serum, stool, gastric aspirates, or suspected food sources (Rao et al. 2021). However, the slow turnaround of results—often several days—means that antitoxin treatment should not be delayed pending confirmation. In suspected outbreaks, public health authorities are typically involved to coordinate testing and containment strategies.

## Current treatment of botulism

Botulism is a medical emergency in which the timing of intervention significantly affects the outcome (Rao et al. 2021). While the mechanisms behind BoNT-induced flaccid paralysis are understood, current treatments are unable to reverse paralysis in already affected neurons. Thus, the focus of treatment is on stopping the spread of the toxin, maintaining essential bodily functions, and reducing complications during the extended recovery period (Rao et al. 2021; O’Brien et al. 2025). This section outlines the critical components of current botulism therapy, including antitoxin administration, supportive strategies for autonomic dysfunction, and considerations related to antitoxin composition and historical development. It further explains why, despite its theoretical appeal, vaccination remains an impractical public health tool.

Prompt clinical suspicion of botulism and swift administration of botulinum antitoxin are crucial in reducing the consequences of BoNT. The currently available antitoxins are effective in neutralizing circulating BoNT in the bloodstream, halting disease progression, and preventing further paralysis. However, internalized BoNT in neurons is not accessible to current antitoxins, and consequently, they are unable to reverse existing paralysis. Health guidelines, including the Centers for Disease Control and Prevention (CDC), recommend administering the antitoxins within the first 48 h of the initial clinical presentation (Rao et al. 2021). Some health agencies, such as those in Germany, only administer the antitoxin within 24 h after oral ingestion of the toxin (Hellmich et al. 2018). However, the benefit of antitoxin decreases substantially as time passes, particularly once more toxin has entered neurons. Because of the rarity of botulism, healthcare systems—including national public health agencies such as the CDC—distribute antitoxin only upon clinical request and consultation. Furthermore, due to the potential of BoNT to be weaponized as a bioterrorism agent, national defense agencies, including military medical units and emergency preparedness organizations, maintain strategic stockpiles of antitoxin as part of biosecurity protocols (Thirunavukkarasu et al. 2018).

Patients requiring intensive care—especially those with delayed antitoxin administration—often need mechanical ventilation due to respiratory muscle paralysis. Weaning can be prolonged because of the slow recovery of neuromuscular transmission. Prompt respiratory support should be initiated when indicated, as delays could lead to unnecessary complications. Supportive care focuses on preventing ICU complications such as ventilator-associated pneumonia, tracheostomy infection, and pressure ulcers (Rao et al. 2021; Thirunavukkarasu et al. 2018).

Equally important is the management of autonomic nervous system dysfunction in the ICU, which can be severe and persistent. Regular dental hygiene and the use of artificial tears can help mitigate the negative consequences associated with xerostomia (dry mouth) and keratoconjunctivitis sicca (dry eyes), including mucosal damage, corneal abrasion, and secondary infections (Manfredi et al. 2011). Severe constipation is a prominent consequence of parasympathetic dysfunction and typically necessitates a combination of prokinetic medications, stool softeners, and manual interventions (Jenzer et al. 1975; Merz et al. 2003). Ongoing tachycardia might indicate increased sympathetic activity and could lead to cardiac conditions like Takotsubo-like myocardial dysfunction, which may require the use of sympatholytic drugs such as propranolol (Tonomura et al. 2017). It is crucial to avoid anticholinergic medications—including atropine, certain antihistamines, tricyclic antidepressants, and antipsychotics—as they can exacerbate existing autonomic impairments and worsen paralytic symptoms.

Antitoxin therapy remains the only specific pharmacological intervention for botulism. Two main types are currently in use. The first, Heptavalent Botulinum Antitoxin (HBAT), is an equine-derived product that contains F(ab’)2 and Fab fragments targeting BoNT serotypes A through G. The second is Botulism Immune Globulin Intravenous (BabyBIG®), a human-derived antitoxin approved for the treatment of infant botulism due to serotypes A and B. BabyBIG demonstrates superior tolerability and reduced risk of hypersensitivity reactions compared to equine products, but is not used in adults (Rao et al. 2021).

Concerns about the potential use of BoNT as a biological weapon during World War II prompted the U.S. Army military biodefense programs to initiate extensive antitoxin development. The outcome of those efforts was the development of polyvalent equine antitoxins, which were later adapted for civilian use. The current HBAT was developed through a collaboration between the U.S. Army and the CDC and was licensed for use in 2013. Unlike earlier whole immunoglobulin formulations, HBAT is composed of F(ab’)2 fragments, thereby reducing immunogenicity and the risk of serum sickness. Its production involves immunizing horses with detoxified BoNT toxoids, followed by plasma harvesting, pepsin digestion, and affinity purification to isolate the desired immunoglobulin fragments (Rao et al. 2021; Arnon et al. 2001).

Although a vaccine has been available since the 1970 s, it is not routinely administered to civilians. A pentavalent botulinum toxoid vaccine was created for military and laboratory staff, but it is not commercially available due to limited demand, the rarity of botulism in the general population, and the requirement for multiple doses to achieve temporary protective immunity (Liu et al. 2024; Gupta and Pellett 2023). Furthermore, the vaccine requires complex production methods and cold-chain logistics, making it unsuitable for mass immunization. The potential for immunological cross-reactivity with therapeutic BoNTs used in clinical practice (e.g., in dystonia or cosmetic procedures) also raises concern. Consequently, vaccination is now limited to high-risk occupational groups under investigational new drug (IND) protocols (Liu et al. 2024; Wang et al. 2025; Qiaerxie et al. 2024).

In conclusion, mechanical ventilation has dramatically reduced the mortality rate of botulism from 85–90% to less than 10%. Current management should prioritize early recognition and prompt antitoxin administration to avoid prolonged ICU admission and related complications. Supportive care plays an equally vital role, particularly in managing autonomic complications and preventing secondary sequelae of prolonged paralysis. Given the limited therapeutic options, substantial research and development are still needed to develop treatments capable of neutralizing BoNT after it has been internalized in neurons. Meanwhile, timely clinical suspicion remains the most powerful tool against this formidable neurotoxin.

## Preclinical therapeutic interventions for botulism

BoNT toxicity can be devastating for patients and may rapidly strain healthcare systems due to the urgent need for ICU care and mechanical ventilators. Preclinical research on BoNT faces several challenges. The extreme potency of BoNT (LD_50_ measured in nanograms) requires high-containment facilities (BSL-3 or higher), while animal research involving BoNT frequently faces considerable ethical scrutiny because of the neuroparalytic effects and the lasting symptoms of botulism. As a result, the processes of screening, validating, and achieving reproducibility of potential treatments for BoNT remain challenging (Patel et al. 2022; Lin et al. 2020). An overview of current and emerging therapeutic modalities against BoNTs is summarized in Fig. 3. This figure maps the spectrum of interventions, ranging from conventional antitoxin therapy to novel strategies such as receptor-binding inhibitors, LC degradation strategies, and gene/RNA-based approaches, thereby framing the discussion of experimental countermeasures.

Despite these challenges, the severe and persistent neuromuscular paralysis induced by BoNTs—primarily through cleavage of synaptic SNARE proteins—underscores the urgent need for effective therapeutic interventions. Spontaneous recovery may require weeks to months because of the prolonged intracellular persistence of the toxin LC, prompting interest in methods to expedite BoNT clearance or inhibit its mechanism of action. Preclinical therapeutic approaches fall into six main categories: (1) neutralization of circulating toxin with antitoxins or monoclonal antibodies; (2) enzymatic inhibition that targets the LC protease; (3) enhancement of intracellular toxin clearance, including ubiquitin–proteasome-mediated degradation; (4) prevention of cellular entry by interfering with receptor binding or endocytosis; (5) gene-based therapies, such as the delivery of cleavage-resistant SNAP-25 mutants; and (6) RNA-based approaches. These therapeutic modalities, along with their primary mechanisms, developmental stages, key advantages, and major limitations, are summarized in Table 2 for a comparative overview. Each approach is discussed in detail below.

**Table 2:** Comparative summary of emerging and current therapeutic modalities for botulism. The table highlights primary mechanisms, current developmental stage, main advantages, and principal limitations of each approach

Therapeutic Modality	Primary Target/Mechanism	Stage of Development	Key Advantages	Major Limitations
Clinical antitoxins (HBAT, BabyBIG®)	Antibody-mediated neutralization of circulating BoNT via binding to the heavy chain (HC)	Clinical (approved)	Established clinical efficacy; rapid neutralization of extracellular toxin; reduces morbidity and mortality when administered early	Ineffective once toxin is internalized; narrow therapeutic window; equine-derived products may cause hypersensitivity reactions
Next-generation antibodies (recombinant mAbs, multi-epitopic antibodies, VHHs)	High-affinity neutralization of extracellular BoNT through mono- or multi-epitope binding	Preclinical (in vivo)/Early clinical (selected candidates)	Improved scalability and reduced immunogenicity; potential multi-serotype coverage; suitable for prophylaxis and biodefense	Limited to extracellular toxin; manufacturing complexity for multi-component formats; short half-life of VHHs unless engineered
Small-molecule inhibitors (LC protease or HC-mediated entry)	Inhibition of LC enzymatic activity or interference with HC-dependent receptor binding/translocation	Preclinical (in vitro and in vivo)	Theoretical post-internalization activity; amenable to structure-guided optimization; broad chemical diversity	Limited translation from in vitro potency to in vivo efficacy; challenges in neuronal delivery, pharmacokinetics, and serotype specificity
Intracellular clearance strategies (DUB inhibitors, PROTAC-based approaches)	Promotion of LC degradation via modulation of the ubiquitin–proteasome system	Early preclinical (primarily in vitro proof-of-concept)	Directly targets intracellular toxin persistence; event-driven pharmacology; potential application as safety switches for engineered BoNTs	Immature for clinical translation; substantial delivery barriers to motor neurons; risk of off-target proteostasis effects
Gene- and RNA-based therapies (AAV-delivered resistant SNAREs, intrabodies, mRNA-encoded antibodies)	Intracellular expression of toxin-resistant proteins or neutralizing biologics	Preclinical (in vivo)	Potential for durable or single-dose prophylaxis; restoration of neuronal function; rapid scalability of mRNA platforms	Unsuitable for acute intervention; delivery and immunogenicity challenges; long-term safety and regulatory uncertainty
Vaccines (recombinant subunit, chimeric, nucleic acid platforms)	Prophylactic induction of host neutralizing antibodies against non-toxic BoNT domains (e.g., Hc)	Preclinical (in vivo)/Historical IND (PBT)	Preventative strategy; suitable for high-risk populations; recombinant platforms improve scalability and safety	Not therapeutic; requires priming and boosting; potential interference with therapeutic BoNT applications

### Neutralizing antibodies and antitoxins

The only clinically proven therapy for botulism is antibody-based neutralization targeting the receptor-binding domain of BoNT. A key limitation is timing: antibodies are only effective while BoNT remains in circulation prior to neuronal internalization. Currently available products include equine HBAT and human immunoglobulin BabyBIG®, which neutralize circulating toxin in the bloodstream (Rao et al. 2021; Arnon et al. 2006). Despite their effectiveness, these antitoxins face supply and safety constraints during high demand. They are produced using non-recombinant methods that rely on live immunization, making manufacturing labor-intensive and difficult to scale. In addition, equine-derived antitoxin can trigger hypersensitivity reactions (Lou et al. 2025; Black And Gunn 1980). These limitations have driven the development of newer antibody formats to improve scalability and reduce immunogenicity.

Recombinant monoclonal antibodies (mAbs) could address several limitations of traditional antitoxins. They may also be useful for prophylaxis in high-risk individuals and as part of a biodefense preparedness against BoNT (Snow et al. 2021). Early studies using a single mAb showed limited efficacy (Nowakowski et al. 2002). In contrast, combinations of mAbs that bind non-overlapping epitopes can act synergistically and improve neutralization (Lou et al. 2025). This likely reflects more complete blockade of distinct functional sites on the toxin. Several antibody mixtures have shown promising protection in animal models and are advancing clinically. For example, XOMA 3AB (three mAbs against BoNT/A) provided protection in primate models, and G03-52–01 (a six-IgG product targeting BoNT/A and BoNT/B) demonstrated efficacy as pre-exposure prophylaxis against inhalational botulism in guinea pigs and is under clinical evaluation (Snow et al. 2021; Lou et al. 2025; Nayak et al. 2014). However, multi-antibody approaches introduce substantial manufacturing and bioanalytical complexity, because multiple distinct antibodies must be produced and co-formulated.

An innovative solution is the development of multi-epitopic antibodies, which are single, engineered IgG-based molecules that incorporate the variable domains of multiple parental mAbs. This approach aims to reproduce the potency of a mixture of several mAbs within a single antibody, which may simplify both production and the associated bioanalytical tasks. The potential of this approach has been demonstrated in several preclinical studies. Lou and others developed a tri-epitopic antibody (TeAb) against BoNT/A, BoNT/B, BoNT/E, and BoNT/F, which demonstrated similar efficacy compared to the three mAbs combination in the mouse neutralization assay (MNA) (Lou et al. 2025).

Another approach is to use camelid-derived nanobodies, also known as variable domains of heavy-chain-only antibodies (VHHs), as antitoxins. Compared with traditional antibodies, nanobodies are small (~ 15 kDa), can penetrate tissues more readily, are stable, and are easy to engineer, while maintaining low immunogenicity and the ability to bind challenging epitopes (Godakova et al. 2019; Lam et al. 2020). In animal studies, VHH-based antitoxins have shown strong protection against BoNT (Quynh Pham et al. 2025; Tremblay et al. 2020; Lam, et al. 2020). A key limitation is their short half-life, which can be improved by fusion to an IgG Fc region (Godakova et al. 2019). Overall, VHHs may reduce some manufacturing and immunogenicity challenges seen with earlier antitoxins. However, like other antibody-based antitoxins, they mainly neutralize extracellular toxin and do not address BoNT already internalized within neurons. This is why newer work focuses on strategies that block or remove the toxin after neuronal entry.

Structural biology has directly enabled the rational development of these next-generation countermeasures by providing atomic-level insights into toxin-antibody interfaces. High-resolution crystal structures of BoNT/A1 and BoNT/B1 in complex with VHHs have mapped critical neutralizing epitopes within the receptor-binding domain. By defining the precise coordinates of these footprints, researchers identified non-overlapping binding sites that can be targeted simultaneously. This structural mapping guided the engineering of heterodimeric VHH-based neutralizing agents (VNAs) joined by flexible linkers, which facilitate the concurrent engagement of two distinct epitopes on a single toxin molecule. These bifunctional VNAs leverage a ‘chelate effect’ to achieve superior binding kinetics, providing robust in vivo protection against lethal challenges and bridging the gap from molecular understanding to translational efficacy.

### Intracellular clearance strategies for bont light chain

The catalytic LC of BoNT exerts its pathogenic effect by cleaving SNARE proteins, which are essential for synaptic vesicle fusion and neurotransmitter release. Therefore, blocking or eliminating LC protease activity represents a druggable target for post-exposure therapies (Caglic et al. 2014). Initial investigations concentrated on hydroxamate-based metalloprotease inhibitors; however, these agents were limited by poor cellular uptake and metabolic instability (Thompson et al. 2011; Jacobson et al. 2017; Lin et al. 2023). More recently, high-throughput screening and structure-guided design have produced candidates with better potency. For example, quinolinol- and benzimidazole-based derivatives inhibit LC activity in *vitro* at nanomolar concentrations. However, despite strong in *vitro* activity, these small molecules showed limited benefit in animal models, likely due to poor target engagement in neurons (Lin et al. 2023).

An alternative approach aims to leverage the host ubiquitin–proteasome system to degrade the intracellular LC (Tsai et al. 2010; Kuo et al. 2011). Compounds such as PR-619 and WP1130, which target deubiquitinating enzymes (DUBs), have shown the ability to destabilize the BoNT LC by preventing the removal of ubiquitin tags, thereby facilitating proteasomal breakdown. These compounds can alleviate the neurotoxic effects induced by BoNT, even after the toxin has been internalized in several in *vitro* models of BoNT toxicity (Sen et al. 2021).

A more recent and sophisticated strategy to enhance the clearance of the BoNT LC involves leveraging proteolysis-targeting chimeras (PROTACs) (Tsai et al. 2024). PROTACs are bifunctional small molecules: one moiety binds the target protein and the other recruits an E3 ubiquitin ligase, leading to ubiquitination and proteasomal degradation of the target (Sakamoto et al. 2001; Buckley et al. 2015). Tsai et al. demonstrated this concept using a fusion construct of mFKBP (a ligand-responsive domain incapable of recruiting E3 ligases on its own) and VHH B8, a camelid nanobody that binds BoNT/A LC and inhibits its enzymatic activity. When cells co-expressed mFKBP–VHH B8 with GFP-tagged BoNT/A LC, treatment with the mFKBP-specific PROTAC dTAG V-1 caused dose-dependent destabilization of the LC. Although this study provides an initial proof-of-concept for targeted LC degradation using an engineered system, its clinical relevance remains conceptual. It suggests that if recombinant BoNTs were engineered with PROTAC-compatible tags for therapeutic use, a corresponding PROTAC molecule could theoretically be administered as a ‘safety switch’ in the event of accidental overdose. However, these preliminary in *vitro* studies require validation in robust in vivo models (Tsai et al. 2024).

### Small-molecule inhibitors targeting bont domains

Given the limitations of antibody-based therapies, considerable interest has focused on small molecules that can access neurons and directly target BoNT. These molecules may offer the advantage of cellular access, enabling designs that focus on disrupting the multistage pathogenic process of BoNT (Ben David et al. 2021). Mechanistically, small-molecule inhibitors fall into four categories: (1) those targeting the LC catalytic (protease) domain, (2) those blocking HC-facilitated receptor binding and/or translocation, (3) dual-targeting or multifunctional agents, and (4) innovative compounds with unclear or hybrid modes of action (Patel et al. 2022; Ben David et al. 2021; Lin et al. 2025; Machamer et al. 2022; Bremer et al. 2017; Bremer et al. 2016). Each strategy brings its own set of pharmacological benefits and hurdles in translation to clinical use, as evidenced by the evolving body of preclinical research.

The LC of BoNT acts as a zinc-dependent metalloprotease that cleaves SNARE proteins, which are critical for synaptic vesicle fusion and neurotransmitter release. This proteolytic action prevents the release of ACh at the NMJ and autonomic synapses, which clinically manifests as the life-threatening flaccid paralysis characteristic of botulism (Bach And Simman 2022). Because this enzymatic activity is the direct cause of toxicity, the LC has become a primary druggable target for post-exposure therapeutic development. Advances in structural biology have shifted the development of these small molecules from empirical screening to mechanism-based design. High-resolution crystallographic analysis of the LC catalytic cleft uncovered the plastic nature of the zinc-dependent metalloprotease core and identified distinct substrate-recognition exosites. For example, the characterization of a hydrophobic cleft adjacent to the active site and the mapping of the β-exosite enabled the rational design of inhibitors such as 5-substituted picolinic acids and DCHA derivatives. By exploiting specific interactions—such as hydrogen bonding with Tyr250 or extending alkyl side chains to increase active-site residence time—these structure-guided modifications achieved significantly improved potency and metabolic stability compared to generic zinc chelators. The key rationale for this approach is that small-molecule inhibitors may be able to cross neuronal membranes and neutralize the toxin after internalization.

Several laboratories have employed high-throughput screening techniques and rational design using structure–activity relationship studies to develop small-molecule drugs against BoNT. These small molecules were directed at the active site, the α-exosite, or the β-exosite binding sites of BoNT/A LC. For instance, Janda and colleagues have published multiple research articles describing small molecules with numerous mechanisms of action against the BoNT/A LC (Lin et al. 2020; Fischer et al. 2009). While several chemicals demonstrated excellent pharmacodynamic properties in biochemical and cell-based assays, they performed poorly in mouse bioassay evaluations. This disconnect may be explained by factors such as inadequate cell permeability, rapid metabolic clearance, and unintended effects resulting from conserved Zn^+2^ binding motifs. Furthermore, some compounds demonstrated non-specific reactivity in vivo or did not sufficiently engage with the target. Additionally, numerous reports from various research groups have highlighted small molecules with outstanding in *vitro* results; however, these have either not been tested in vivo or have shown modest performance in animal studies (Lin et al. 2020; Lin et al. 2025).

Atomic-level insights have similarly expanded the chemical toolbox to include irreversible inactivation strategies, addressing the limitations of earlier reversible inhibitors. X-ray co-crystal structures of the BoNT/A LC identified a non-catalytic Cys165 residue positioned near the active-site Zn. This structural insight enabled the synthesis of bifunctional inhibitors that use a metal-binding group to anchor the molecule at the zinc site while orienting a covalent warhead toward the deep-seated Cys165. This orientation helps overcome the challenges posed by residue burial and low nucleophilicity, resulting in selective, irreversible inhibition. Furthermore, structural analysis of the translocation domain inspired the identification of channel inhibitors, such as toosendanin analogs, which arrest the toxin during its transition from the endocytic vesicle to the cytosol.

Small-molecule inhibitors that target the HC domain of BoNT have surfaced as a viable therapeutic approach to prevent neuronal intoxication by obstructing receptor binding or membrane translocation. Recent research has pinpointed compounds that disrupt the interactions between gangliosides or protein receptors essential for BoNT internalization, with several showing efficacy. These inhibitors, which include quinolinol derivatives and molecules that mimic receptors, have displayed potential in both cell-based assays and in vivo systems, particularly against serotypes A and B (Ben David et al. 2021; Dhaked et al. 2010). Nevertheless, the advancement of HC-targeting agents continues to be challenged by structural differences among serotypes, the conformational flexibility of the binding interface, and the absence of high-throughput screening platforms specifically designed for translocation inhibition.

### Gene and RNA-based therapies

Gene and RNA-based strategies have recently emerged as innovative approaches for countering BoNT intoxication. Unlike conventional antitoxin therapies, which rely on neutralizing circulating toxin, nucleic acid–based approaches aim to directly restore or protect neuronal function at the molecular level. Several complementary strategies are under investigation, including delivery of toxin-resistant SNARE proteins (Raghunath et al. 2008), expression of intracellular antibodies (intrabodies) (McNutt et al. 2021), and RNA-based technologies designed to neutralize or bypass the catalytic activity of the toxin LC (Panova et al. 2023).

One of the earliest demonstrations of feasibility involved the use of adeno-associated viral (AAV) vectors to deliver a cleavage-resistant variant of SNAP-25. Expression of this mutant protein (S25-R198T) in chromaffin cells preserved exocytosis following BoNT/A challenge, and in vivo administration into the rat spinal cord attenuated neuromuscular paralysis and reduced nerve sprouting typically associated with intoxication. These findings established proof-of-concept that gene replacement strategies could substitute for toxin-truncated proteins and maintain neurotransmission (Raghunath et al. 2008). In parallel, viral and non-viral vectors have been used to deliver single-chain antibodies or intrabodies directed against BoNT LC. Sustained expression of such intrabodies within motor neurons provided partial protection in rodent models, highlighting their potential as long-term prophylactic tools. However, translation is constrained by concerns over vector immunogenicity, limited transduction efficiency of motor neurons, and uncertainties regarding long-term safety (Panova et al. 2023).

Building on these foundations, RNA-based strategies are gaining momentum, particularly with the success of lipid nanoparticle (LNP)–formulated mRNA vaccines. Recent studies have demonstrated that mRNA encoding recombinant neutralizing antibodies can achieve rapid in vivo expression and confer protection equivalent to that of protein-based antibodies. For example, mRNA encoding a camelid VHH-Fc fusion (B9-hFc) produced sustained protective antibody titers in mice and effectively prevented BoNT/B intoxication following a single LNP injection (Qiaerxie et al. 2024). Similarly, replicating RNA (repRNA) platforms have been engineered to encode long nanobody heterohexamers, enabling simultaneous neutralization of multiple serotypes (A, B, and E). Intramuscular administration of formulated repRNA induced rapid antitoxin expression, detectable within hours, and protected mice against supralethal challenges, demonstrating both breadth and potency (Mukherjee et al. 2022). Beyond antibody expression, RNA aptamers and antisense oligonucleotides have also been explored to suppress LC expression or inhibit its catalytic activity (Chang et al. 2010; Chang et al. 2016). While these approaches remain preclinical, they offer a conceptual framework for designing molecules that directly target the enzymatic machinery of BoNT.

Collectively, these studies underscore that gene and RNA-based interventions provide a distinct therapeutic paradigm: rather than neutralizing toxin extracellularly, they aim to restore neuronal function or generate protective molecules de novo within the host. Nevertheless, major translational barriers remain. AAV-based therapies face challenges related to dosing, serotype selection, and long-term immunogenicity, while RNA-based systems require optimization of delivery, stability, and duration of expression. Importantly, these modalities are more suited to prophylaxis or post-exposure scenarios with extended therapeutic windows, rather than acute treatment where rapid neutralization is essential.

Despite these limitations, the convergence of advanced vectorology, mRNA platforms, and nanobody engineering positions gene- and RNA-based therapies at the frontier of BoNT countermeasures. They hold particular promise for high-risk populations, biodefense applications, and scenarios where durable, broad-spectrum protection is required. As such, these strategies may ultimately complement traditional antibody- and small-molecule–based approaches, forming a multi-layered arsenal against botulism.

### Vaccine approaches in preclinical development

Vaccination remains a cornerstone strategy in protecting against BoNTs, particularly in populations at occupational or military risk. The pentavalent botulinum toxoid (PBT), developed in the mid-twentieth century, was historically administered under investigational protocols by the CDC. This inactivated toxoid provided broad coverage against serotypes A–E and demonstrated a long-standing safety profile. However, its application was restricted to defined high-risk groups, such as military personnel and laboratory workers directly handling BoNT, due to several inherent limitations. These included a cumbersome production process reliant on formaldehyde detoxification, batch-to-batch variability, a requirement for repeated dosing, and the waning of protective immunity over time (Abdolmohammadi Khiav And Zahmatkesh 2022). The CDC’s discontinuation of PBT distribution for laboratory workers in 2011 underscored the need for safer, more scalable alternatives.

The advent of recombinant DNA technology has enabled the generation of second-generation vaccines designed to overcome these shortcomings. Recombinant subunit vaccines, in particular, exploit the receptor-binding domain of the BoNT HC, which is non-toxic yet immunogenic. Preclinical studies have consistently demonstrated that Hc-based vaccines elicit neutralizing antibodies and confer protection across multiple animal models, including mice, guinea pigs, and non-human primates (Shi et al. 2020; Shi et al. 2022). Notably, recombinant BoNT/E Hc (rEHc) elicited robust immune responses in mice, supporting its development as a candidate vaccine. Similarly, a tetravalent formulation incorporating Hc fragments from serotypes A, B, E, and F demonstrated strong immunogenicity, stability over prolonged storage, and complete protection in murine challenge studies (Shi et al. 2022). Beyond Hc, alternative antigenic fragments have been explored. The LC–translocation domain (L–HN) construct of BoNT/E has shown superior protective efficacy compared with Hc alone, suggesting that broader domain coverage may present additional neutralizing epitopes while maintaining safety (Li et al. 2022). Furthermore, chimeric and multivalent vaccine platforms have gained traction. For example, a streptavidin–biotin conjugation system has been engineered to generate a trivalent BoNT/A–B–E vaccine, which produced high-titer neutralizing antibodies and complete protection against lethal toxin challenge in mice (Liu et al. 2024). These platforms provide both antigenic breadth and enhanced structural stability, addressing challenges in scalability and long-term storage.

Adjuvant optimization represents a parallel area of innovation. Aluminum salts remain the most widely used adjuvant, but novel formulations such as saponin-based adjuvants are being investigated to improve both the magnitude and durability of neutralizing antibody responses (Gupta and Pellett 2023). Importantly, adjuvant choice may reduce the number of doses required, a key advantage for biodefense applications where rapid and durable immunity is paramount.

While recombinant subunit vaccines dominate the preclinical landscape, emerging approaches also include DNA- and mRNA-based vaccines encoding non-toxic BoNT domains. These strategies leverage rapid production and scalability, as demonstrated during the COVID-19 pandemic, but remain in early development stages for BoNTs (Gupta and Pellett 2023). Their ability to induce both humoral and cellular immunity makes them attractive for future evaluation in biodefense contexts.

Collectively, these advances highlight a clear trajectory away from classical toxoids toward recombinant and engineered vaccines. Although none are yet licensed for general human use, the expanding pipeline of subunit, chimeric, and nucleic acid-based candidates underscores the progress toward safe, effective, and deployable BoNT vaccines. Future directions should prioritize multivalent coverage, optimized adjuvantation, and scalable production systems to ensure preparedness against both natural outbreaks and deliberate misuse of these Category A biothreat agents (Avril et al. 2024).

## Barriers to clinical implementation

Despite the rapid expansion of preclinical countermeasures against BoNTs, clinical translation remains constrained by recurring barriers that span safety, intracellular delivery, immunogenicity, and real-world deployment logistics. These limitations are particularly pronounced for post-exposure interventions, where the therapeutic window narrows sharply once the toxin has entered neurons and the LC becomes sequestered in the cytosol. In this context, clinical implementability depends not only on mechanistic efficacy, but also on whether a modality can be delivered safely, at scale, and within regulatory and biodefense constraints.

A primary barrier is toxicity and tolerability, which differ by modality but remain central to regulatory viability. For small-molecule approaches, strong biochemical potency often fails to translate in vivo because achieving meaningful exposure at the neuromuscular junction may require systemic dosing that increases off-target risk, particularly for scaffolds that rely on metal-binding motifs or broadly-reactive chemotypes. This alignment problem has repeatedly emerged in LC-directed inhibitor programs, where cellular activity and in vivo protection often fail to correlate with in *vitro* performance, reinforcing the need for early de-risking of selectivity, distribution, and metabolism (Caglic et al. 2014; Bremer et al. 2017). Host-directed strategies that modulate proteostasis or ubiquitin signaling introduce an additional safety layer. The therapeutic goal is to shorten LC persistence without perturbing essential neuronal ubiquitin–proteasome functions, which are tightly coupled to neuronal survival and synaptic homeostasis (Tsai et al. 2024; Kuo et al. 2011).

A second, cross-cutting barrier is delivery for intracellular targeting. Neutralizing antibodies and many receptor-binding antagonists operate extracellularly and therefore cannot reverse established intracellular intoxication, even if they remain valuable for early treatment and prophylaxis (Avril et al. 2024). In contrast, LC-directed inhibitors, degradation strategies, and intracellular binders must reach motor neurons, traverse relevant membranes, and accumulate in the same intracellular compartment as the toxin. Proof-of-concept intracellular clearance strategies—including targeted ubiquitination and PROTAC-enabled degradation of LC—underscore the promise of intracellular intervention, but they also highlight the translational friction created by cell entry requirements, tissue distribution, and the need for adequate presynaptic exposure without systemic liability (Tsai et al. 2024; Kuo et al. 2011). For nucleic-acid platforms, delivery is similarly decisive: the platform must achieve efficient expression in vivo while maintaining tolerability and predictable biodistribution, and the formulation constraints can become rate-limiting as programs move from animal challenge studies to scalable clinical products (Panova et al. 2023; Mukherjee et al. 2022).

A third barrier is immunogenicity and long-term safety for gene/RNA-based strategies. Viral-vector approaches face well-recognized hurdles, including pre-existing anti-vector immunity, dose limitations, and the possibility that immune responses constrain repeat dosing or durability—issues that complicate both clinical trial design and biodefense use cases (Panova et al. 2023). RNA platforms can enable rapid in vivo expression of neutralizing biologics and may better match surge-response needs; however, they still require careful management of innate immune activation, reactogenicity, durability of expression, and repeat-dose feasibility (Panova et al. 2023; Mukherjee et al. 2022). Importantly, these immunologic considerations are not merely theoretical: for emergency deployment, a countermeasure must remain effective across heterogeneous populations with variable baseline immunity and must demonstrate a safety margin compatible with use outside highly specialized centers(Gupta and Pellett 2023).

Finally, scalability and biodefense readiness impose operational constraints that are not captured by standard efficacy endpoints. Antitoxin and antibody strategies raise practical questions of manufacturing throughput, lot-to-lot consistency, cost, cold-chain requirements, and stockpiling stability—particularly when protection depends on oligoclonal mixtures or multi-component formats (Snow et al. 2021; Avril et al. 2024). Vaccine and nucleic-acid platforms may offer advantages in speed of design and scale-up, but they introduce their own deployment trade-offs, including specialized formulation, storage, and distribution requirements, as well as the need for validated large-scale manufacturing workflows that can be activated rapidly during an emergency (Liu et al. 2024; Gupta and Pellett 2023). From a biodefense standpoint, the most deployable countermeasures will be those that combine broad serotype coverage with feasible manufacturing and storage profiles, enabling surge distribution without compromising product quality (Avril et al. 2024).

Collectively, these barriers explain why multiple BoNT countermeasures remain preclinical despite substantial mechanistic progress. Bridging the translational gap will likely require coordinated optimization across modalities: (i) improved safety and target engagement for small molecules, (ii) delivery solutions that reliably access intoxicated motor neurons, (iii) immunogenicity-aware development pathways for gene/RNA platforms, and (iv) manufacturing and logistics strategies aligned with biodefense stockpiling and rapid deployment (Gupta and Pellett 2023).

## Conclusion and future directions

The complexity of BoNT toxicity, including its prolonged intracellular persistence and sequential mechanism of action, necessitates the exploration of multiple therapeutic strategies. Currently, the main clinical approach for treating BoNT is antibody-based therapy. However, its success is contingent on timely administration, and it is ineffective against intracellular toxin, highlighting a significant therapeutic gap. Numerous preclinical studies have investigated various therapeutic strategies— including inhibiting intracellular BoNT activity, modulating host cell degradation processes, exploring gene-based approaches, and utilizing receptor-binding antagonists— with varying degrees of success. Collectively, these strategies may broaden the therapeutic window for targeting intracellular BoNT. However, several translational challenges remain, including the necessity to refine intracellular delivery methods, improve pharmacokinetic properties, and confirm therapeutic efficacy across a range of serotypes. Advancing this field will require not only therapeutic innovation but also method standardization. Well-established, reproducible animal models, and biomarker-based outcome measures will be essential to enable cross-study comparisons and to support regulatory confidence in therapeutic efficacy. Ultimately, a multidisciplinary approach—combining structural biology, pharmacology, and neurobiology—will be required to bridge the gap between laboratory research and clinical application.

## Data Availability

This article is based on a comprehensive literature review of published research. All data supporting the conclusions of this review are included within the article and its references. No original experimental data were generated for this review. All cited literature sources are publicly available through their respective publishers and databases.

## Declarations

### Generative AI and AI-assisted technologies in the manuscript preparation process

During the preparation of some parts of this manuscript, the author used AI large language models such as ChatGPT and Grok to draft and refine prose from selected author materials. Specifically, the author supplied the tool with manually curated references and author-generated notes/outlines, and the tool was then used to synthesize paragraph-level drafts and improve clarity and readability. All content was subsequently verified, edited, and approved by the author, who takes full responsibility for the accuracy, integrity, and originality of the work.
